# The multifaceted oncogene SND1 in cancer: focus on hepatocellular carcinoma

**DOI:** 10.20517/2394-5079.2018.34

**Published:** 2018-07-10

**Authors:** Saranya Chidambaranathan-Reghupaty, Rachel Mendoza, Paul B. Fisher, Devanand Sarkar

**Affiliations:** 1 Department of Human and Molecular Genetics, Virginia Commonwealth University, Richmond, VA 23298, USA.; 2 Massey Cancer Center, Virginia Commonwealth University, Richmond, VA 23298, USA.; 3 VCU Institute of Molecular Medicine, Virginia Commonwealth University, Richmond, VA 23298, USA.

**Keywords:** Staphylococcal nuclease and tudor domain containing 1, hepatocellular carcinoma, inflammation

## Abstract

Staphylococcal nuclease and tudor domain containing 1 (SND1) is a protein that regulates a complex array of functions. It controls gene expression through transcriptional activation, mRNA degradation, mRNA stabilization, ubiquitination and alternative splicing. More than two decades of research has accumulated evidence of the role of SND1 as an oncogene in various cancers. It is a promoter of cancer hallmarks like proliferation, invasion, migration, angiogenesis and metastasis. In addition to these functions, it has a role in lipid metabolism, inflammation and stress response. The participation of SND1 in such varied functions makes it distinct from most oncogenes that are relatively more focused in their role. This becomes important in the case of hepatocellular carcinoma (HCC) since in addition to typical cancer drivers, factors like lipid metabolism deregulation and chronic inflammation can predispose hepatocytes to HCC. The objective of this review is to provide a summary of the current knowledge available on SND1, specifically in relation to HCC and to shed light on its prospect as a therapeutic target.

## INTRODUCTION

Hepatocellular carcinoma (HCC) is the primary liver malignancy arising from hepatocytes. It is the fifth common cancer in men and the ninth common cancer in women. It is the second leading cause of cancer-related deaths worldwide. A high mortality to incidence ratio of 0.95 reflects its poor prognosis and makes it an important public health burden (Globocan 2012). The main causes of HCC are viral infections like hepatitis B and hepatitis C, chronic alcoholism, obesity, liver cirrhosis and non-alcoholic steatohepatitis (NASH)^[1]^. Treatment options are restricted to liver transplantation, surgical resection and ablation. Chemotherapy for HCC is not very promising. HCC incidence has almost tripled since the 1980s and it is the fastest rising cause of cancer related deaths in the US^[2]^. Increase in rates of obesity and non-alcoholic fatty liver disease (NAFLD) is an important factor for this trend.

HCC is usually diagnosed at advanced stages. Unfortunately, patients with advanced HCC do not have the option of treatments like liver transplant or surgical resection since the liver is damaged beyond rescue at this stage. Advanced HCC is also resistant to standard chemo- and radiotherapy. Sorafenib, regorafenib and nivolumab are the three FDA approved chemotherapy drugs for advanced HCC. The multi-kinase inhibitor sorafenib was approved in 2007 and the SHARP trial showed that it increases overall survival of HCC patients from 7.9 to 10.7 months^[3]^. Regorafenib, a sorafenib analog, was approved in 2016 and increases overall survival from 7.8 to 10.6 months^[4]^. Nivolumab, an immune oncology agent that blocks programmed cell death 1 (PD1), a negative regulator of T-cell activation and response, thus allowing the immune system to attack the tumor, was approved in 2017 for patients who have been previously treated with sorafenib contingent on a successful phase III trial^[5]^. Most of these drugs are expensive, effective in only a small percentage of treated patients, cause side effects and do not provide a promising increase in survival^[6]^. Nivolumab increases overall survival to 13.2 months and has a more durable response^[7]^. But, it is administered intravenously every two weeks and has the same demerits as the other chemotherapy drugs. The limitations of the current available treatment options mandate identification of new regulators of HCC that might be targeted to develop effective therapy.

## STAPHYLOCOCCAL NUCLEASE AND TUDOR DOMAIN CONTAINING 1: A MULTIFUNCTIONAL ONCOGENE

### Structure and activation

**Figure 1.**
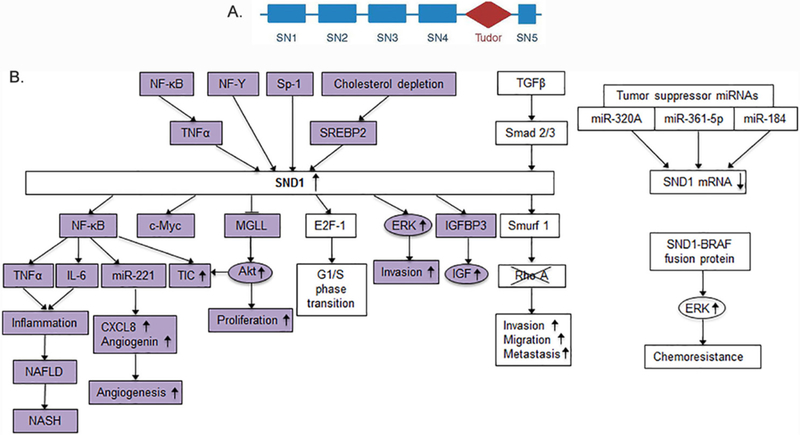
Upstream regulators of SND1 and downstream molecules involved in SND1 activity. A: Structure of SND1 protein; B: schematic overview of the upstream regulators of SND1 and downstream mediators of SND1 activity. Colored molecules indicate those that have been identified in HCC studies. SN: staphylococcal nuclease domains; SND1: staphylococcal nuclease and tudor domain containing 1; HCC: hepatocellular carcinoma

Human staphylococcal nuclease and tudor domain containing 1 (SND1) gene is located at chromosome 7q31.3 and codes for a protein of 910 amino acids with five highly conserved domains. It has a tandem repeat of four staphylococcal nuclease (SN) domains and a fifth fusion domain of a tudor and a partial SN domain [Figure 1A]. SND1 was first identified as a transcription co-activator that interacts with Epstein-Barr nuclear antigen 2 (EBNA2) in lymphocytes^[8]^. It acts as a bridge between the subunits p56 and p34 of the general transcription factor TFIIE and the acidic domain of EBNA2^[9]^. SND1 is an evolutionarily conserved protein in all eukaryotes from protozoa to humans except budding yeast *saccharomyces cerevisiae*^[10–12]^. The upstream regulators of SND1 include the transcription factors NF-κB, NF-Y, Sp1 and SREBP-2 [Figure 1B]. A CpG island with several Sp1 binding sites and an inverted CCAAT box binding to NF-Y regulate basal expression of SND1^[13–15]^. NF-κB binding site is located within the proximal 300 bp segment of SND1 promoter and confers TNFα-mediated induction of SND1^[13]^ [Figure 1B]. SREBP-2 binds to a proximal promoter region containing a serum response element and an enhancer box motif and induces SND1 expression upon cholesterol depletion^[16]^. Activated Smad2 and Smad3 bind to SND1 promoter and confer TGFβ-medicated induction of SND1 expression^[17]^ [Figure 1B].

### Multifaceted properties

Staphylococcal nuclease protects bacteria from invading viruses by degrading viral nucleic acids. In higher organisms, repeats of SN domains and the addition of the tudor domain has created a multifunctional protein in SND1 especially with its ability to interact with a diverse array of proteins. SND1 is involved in regulating gene expression by transcriptional activation^[18–20]^, alternative splicing^[21]^, ubiquitination^[17]^, mRNA stabilization^[22]^ and RNA interference^[23]^. These multifaceted properties allow SND1 to positively impact all hallmarks of cancer, notably sustaining proliferative signaling, evading growth suppressors, resisting cell death, enabling replicative immortality, inducing angiogenesis, and activating invasion and metastasis^[24,25]^.

### Downstream regulators and oncogenic mechanisms

**Figure 2.**
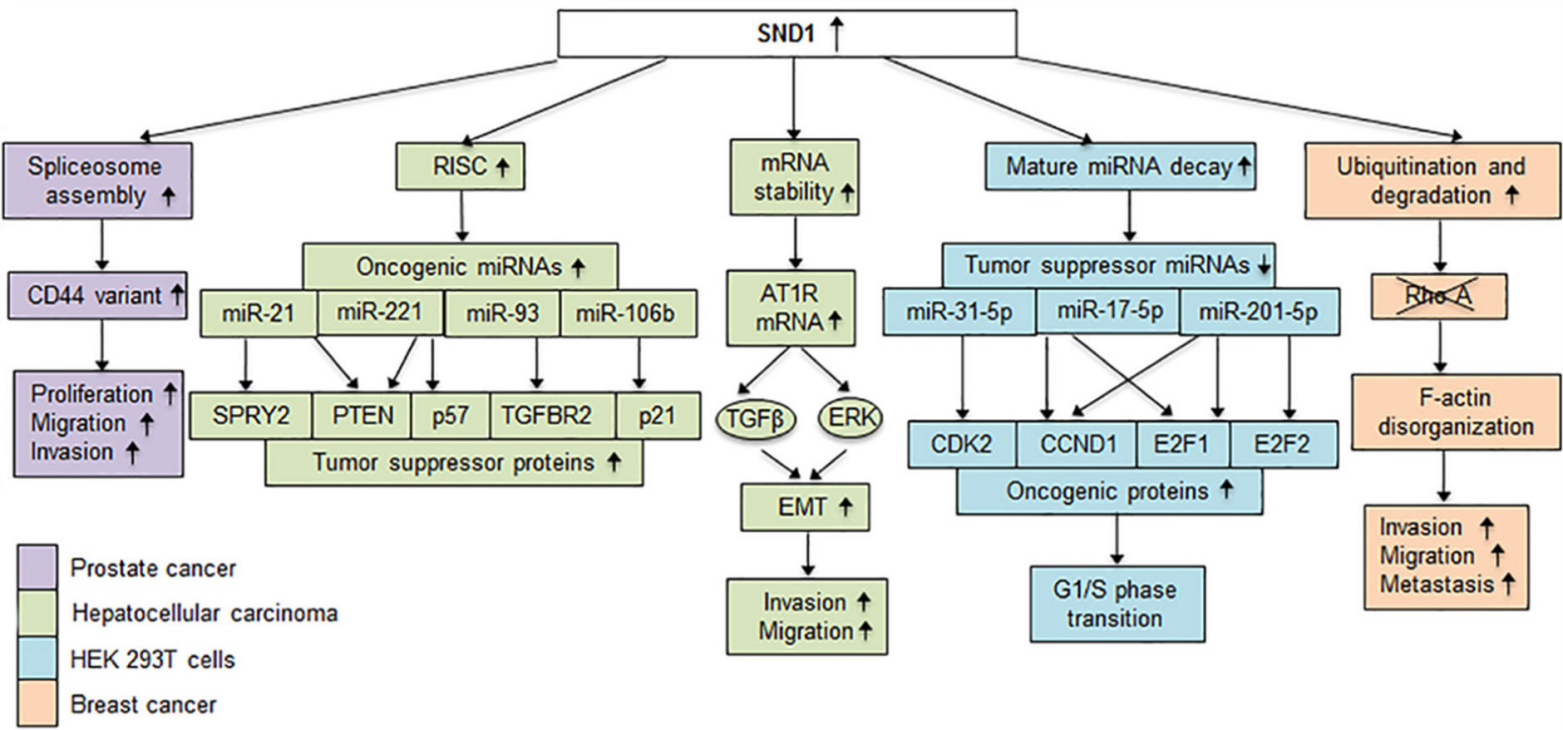
Mechanisms by which SND1 promotes oncogenesis. Downstream molecules that are upregulated, downregulated or degraded due to over expression of SND1 causing a variety of functions to go into disarray leading to tumorigenesis. Each color represents the specific cancer in which the mechanism has been studied. In prostate cancer regulation of spliceosome assembly by SND1 results in the production of an oncogenic variant of CD44 that promotes proliferation, motility and invasion. Tumor suppressor mRNAs that are targets of oncogenic miRNAs are degraded when SND1 over expression confers increased RISC activity in human HCC cells. SND1 increases AT1R mRNA stability, causing an increase in AT1R levels resulting in activation of ERK and TGFβ signaling pathway, promoting EMT and migration and invasion by human HCC cells. SND1 mediates endonucleolytic decay of tumor suppressor miRNAs in HEK293T cells promoting upregulation of oncogenic proteins. In breast cancer cells, SND1 promotes expression of the E3 ubiquitin ligase Smurf1, leading to RhoA ubiquitination and degradation, disrupting F-actin cytoskeletal organization, increasing cell migration and invasion, and promoting metastasis. SND1: staphylococcal nuclease and tudor domain containing 1; HCC: hepatocellular carcinoma

SND1 interacts with and functions as a co-activator for a number of transcription factors that include signal transducer and activator of transcription 5 (STAT5)^[19]^, STAT6^[19,26]^, peroxisome proliferator activated receptor gamma (PPARγ)^[27]^ and c-Myb^[20]^. It functions as a co-activator for the transcription factor E2F-1 facilitating G1/S phase transition^[28]^. SND1 induces the E3 ubiquitin ligase Smurf1 resulting in ubiquitination and degradation of RhoA and promotion of invasion, migration and metastasis^[17]^. SND1 interacts with the U5 spliceosomal RNA to assemble the spliceosome, affecting the levels of various splice variants, such as generation of a variable form of CD44 that promotes motility and invasiveness of prostate cancer cells^[21,29]^. It is a subunit of the RNA-induced silencing complex (RISC) in *caenorhabditis elegans*, drosophila and mammals and functions in miRNA-directed mRNA degradation^[23]^. SND1 is also involved in mature miRNA decay. Knocking out SND1 inhibits cell cycle progression by upregulating a cohort of miRNAs that downregulate mRNAs encoding proteins critical for the G1/S phase transition^[30]^. In parallel to degrading mRNA or miRNA, SND1 shows the ability to bind to 3’-UTR of specific mRNA and increase its stability^[22]^. Transcriptional activation of oncogenes, over-expression of oncogenic splice variants through alternative splicing, degradation of tumor suppressor proteins and silencing of tumor suppressor mRNAs are some of the means used by SND1 to contribute to tumorigenesis [Figure 2]. Given its role in regulating a wide variety of cellular properties, it comes as no surprise that SND1 functions as an oncogene in a variety of cancers, including breast, liver, lung, gastric, glial, prostate and colorectal cancer^[25]^. Although the molecular mechanism by which SND1 is overexpressed in cancer is not clear, it has been identified as a target of a number of tumor suppressor miRNAs, such as microRNA-320a in lung cancer^[31]^, microRNA-361–5p in colorectal and gastric cancer[32], and miRNA-184 in malignant glioma^[33]^ [Figure 1B]. SND1 can be activated by TGF-β1 and in turn activate Smurf1 to promote breast cancer metastasis^[34]^. An SND1-BRAF fusion protein has been identified in gastric, pancreatic and lung cancers that results in activation of downstream MAPK signaling and confers resistance to chemotherapeutic drugs^[35–37]^ [Figure 1B].

## SND1 AS AN ONCOGENE FOR HCC

*In vitro* and *in vivo* studies show that SND1 is an oncogene for HCC. Immunohistochemistry in tissue microarrays containing HCC and adjacent normal liver samples revealed that SND1 is over-expressed in a large percentage (~74%) of HCC patients^[38]^. Chronic inflammation is a critical event in HCC pathogenesis and induction by inflammatory cytokines might underlie the overexpression of SND1 in human HCC patients. Overexpression and knockdown studies in human HCC cells have demonstrated that SND1 promotes proliferation, migration, invasion and *in vivo* tumorigenesis^[38–41]^. As a component of the RISC in HCC cells, SND1 promotes oncogenic miRNA-mediated degradation of tumor suppressor mRNAs^[38]^ [Figure 2]. Some of the mRNAs degraded are PTEN, p57, p21, SPRY2 and TGFBR2 that are targets of miR-221 and miR-21, miR-221, miR-106b, miR-21 and miR-93, respectively^[38]^. These miRNAs are known to be overexpressed in HCC and function as oncogenes. It should be noted that the primary nuclease in the RISC is the argonaute proteins and although a specific small molecule inhibitor of SND1 could partially block RISC activity, SND1 may not be the primary endonuclease in the RISC^[23]^. However, when overexpressed, SND1 could significantly augment RISC activity in human HCC cells when compared to normal hepatocytes^[38]^.

By binding to and stabilizing angiotensin II type 1 receptor (AT1R) mRNA, SND1 activates TGFβ and ERK signaling, thereby promoting epithelial-mesenchymal transition (EMT), *in vitro* migration and invasion by HCC cells^[39]^. In HCC cells, SND1 activates NF-κB, resulting in induction of miR-221 and angiogenic factors angiogenin and CXCL16 that promote tumor angiogenesis^[40]^. Monoglyceride lipase (MGLL) inhibits Akt activation and SND1 interacts with and induces degradation of MGLL, resulting in activation of Akt and subsequent augmentation of cell proliferation and cell cycle progression by human HCC cells^[41]^. SND1 downregulates IGFBP3 expression in human HCC cells that might result in activation of insulin-like growth factor (IGF) signaling, a frequent event in human hepatocarcinogenesis^[42]^.

*In vivo* studies with hepatocyte specific SND1 over-expressing mice (Alb/SND1) showed that transgenic animals have a higher incidence of spontaneous tumors, an increase in CD133+, CD44+ and EpCAM+ tumor initiating cells (TICs) and an increase in HCC drivers (c-Myc, TNFα and IL-6)^[43]^. Upon treatment with a liver carcinogen, diethylnitrosamine (DEN), Alb/SND1 mice showed robustly aggressive tumor response and an increased expression of HCC (AFP and CD36), angiogenesis (CD31) and proliferation (PCNA) markers. Mechanistically, SND1 overexpression activates Akt, ERK, and NF-κB signaling. Inhibitor studies unraveled roles of Akt and NF-κB signaling in regulating SND1-induced increase in TIC while ERK pathway was shown to regulate SND1-induced invasion [Figure 2]. A small molecule inhibitor of SND1, 3’, 5’-deoxythymidine bisphosphate (pdTp), significantly inhibited growth of orthotopic xenografts of human HCC cells in nude mice accompanied by decrease in markers of TIC and inflammation, thereby confirming SND1 as a potential therapeutic target for HCC and utility of pdTp as a therapeutic agent^[43]^.

## SND1 AND INFLAMMATION

HCC initiation and progression are multistep processes. More than 90% of HCCs arise with hepatic injury and chronic inflammation in the background^[44]^. Inflammation is also a hallmark of NASH, a growing public health concern and a major cause of HCC^[45,46]^. Hepatic injury from viral infections, alcohol or high fat diet can cause cell death and the release of molecules called damage associated molecular patterns (DAMPs) that start the inflammatory cascade as a wound-healing response. The transcription factor NF-κB, regulating a diverse array of pro-inflammatory cytokines, chemokines and adhesion molecules, is the single most important molecule causing inflammation. Overexpression of SND1, either in HCC cell lines or in Alb/SND1 mice, resulted in marked activation of NF-κB, and Alb/SND1 mice presented with increased levels of pro-inflammatory cytokines, such as IL-6 and TNFα, thereby providing a link between SND1 and inflammation^[40,43]^. On the other hand, SND1 itself is regulated by NF-κB[13]. Thus a vicious cycle exists where SND1 augments inflammation and the inflammatory process in turn induces SND1 that might cause predisposition to the development of HCC. As yet, the molecular mechanism by which SND1 activates NF-κB remains to be determined. In primary hepatocytes, inhibition of SND1 activity by pdTp not only abrogated LPS-induced nuclear translocation of p65 subunit of NF-κB but also reduced the level of total p65^[43]^. This finding was also observed in human HCC xenografts in nude mice that were treated with pdTp^[43]^. These findings suggest that as a transcriptional coactivator SND1 might be involved in regulating the expression of p65 itself, a hypothesis that needs to be interrogated.

## SND1 AND STRESS RESPONSE

Under normal physiology, cells respond to stress by activating survival pathways to overcome stress or cell death pathways to eliminate damaged cells. A number of factors determine how cells choose between these two responses, and in the context of cancer a variety of proteins promote cell survival rather than cell death to augment tumorigenesis. SND1 seems to have a role in this stress-induced pro-survival signaling. Cells respond to conditions like oxidative stress, heat shock, viral infection, UV irradiation, DNA damage and hyperosmotic stress by forming stress granules (SGs) that are dense aggregations of translation-stalled mRNAs bound to messenger ribonucleoproteins (mRNPs) in the cytosol. Cancer cells use stress granules as a means to promote survival under adverse conditions of the tumor microenvironment. SND1 was identified as a component of cytoplasmic stress granules formed in response to oxidative stress^[47]^. Ras GTPase activating protein SH3 domain binding protein (G3BP) is a phosphorylation dependent endoribonuclease that assembles stress granules and potentially degrades the SG mRNAs. Under oxidative stress, c-JNK phosphorylates SND1 at threonine 103, promoting the binding of its SN domain with G3BP to form stress granules^[48]^. It is not yet clear if the role of SND1 is limited to assembling these SGs or extends beyond that where the endonuclease activity of SND1 participates in degrading SG mRNAs.

Unfolded protein response or endoplasmic reticulum (ER) stress plays an important role in regulating NASH and NASH-induced HCC^[49]^. ER stress can be simulated *in vitro* by exposing cells to thapsigargin, tunicamycin or ectopic expression of activating transcription factor 6 (ATF6), a crucial transcription factor in the unfolded protein response triggered by ER stress. Simulating ER stress in human liver cancer results in an increase in SND1 promoter activity showing that SND1 has a role in ER stress response^[50]^. However, the functional consequence of this observation is yet to be elucidated. In response to DNA damage, SND1 is recruited to the damage site by Poly ADP-ribose polymerase 1 (PARP-1), a DNA damage sensor^[51]^. The accumulated SND1 recruits to the damage site ATP-dependent chromatin remodeler (ARCA5) and histone acetyltransferase (GCN5), two enzymes that promote chromatin relaxation to enable access of DNA damage response related proteins to the damage site. This results in chromatin relaxation and consequent activation of ATM kinase and downstream DNA repair signaling pathways. Thus SND1 functions as a key determinant providing survival advantage under DNA damage stress.

## ROLE OF SND1 IN LIPID METABOLISM

One of the most important metabolic alterations that occur during tumor development is the deregulation of lipid metabolism. Specifically, lipid biosynthesis rate is increased to provide a survival advantage for tumors. Lipids act as signaling molecules, disrupt normal tissue architecture, promote tumor migration and induce angiogenesis^[52]^. Increased lipid synthesis causes steatogenesis or lipid accumulation, a common feature in carcinomas. In HCC, it is reflected by the formation of cytosolic organelles called lipid droplets (LDs) comprised of a core of neutral lipids coated by amphipathic lipids and associated proteins^[53]^. The role of SND1 in lipid metabolism was evidenced when it was found on the surface of LDs originating from the ER in mammary epithelial cells and adipocytes^[54]^. SND1 interacts with a lipoprotein part of the fatty acid synthase (FASN) complex to form LDs. Under steatogenic conditions, SND1 is targeted from cell compartments like the ER and golgi complex to low density LDs to facilitate their assembly^[55]^.

In addition to lipid storage, SND1 is involved in lipid transport. Once fatty acids are taken up from dietary sources or synthesized in the liver, they are transported to other locations in the body to serve energy demands. Hepatocytes use lipoproteins made up of a non-polar lipid core surrounded by apolipoproteins and amphipathic lipids like phospholipids and cholesterol for this purpose. Though they are structurally similar to LDs, their main function is lipid transport rather than storage. Overexpression of SND1 promotes the secretion of phospholipids that form a part of the lipoproteins in primary hepatocytes and facilitates the transfer of these phospholipids to apolipoproteins before their secretion from hepatocytes^[56]^. Cholesterol is another component of the lipid core in both LDs and lipoproteins, the synthesis of which is regulated by SND1. Under conditions of cholesterol depletion, SREBP2, a regulator of cholesterol uptake and synthesis activates SND1^[16]^. Overexpression of SND1 results in increased cholesterogenesis, metabolically coupled to cholesterol esterification, causing an increase in cholesteryl ester levels^[57]^.

**Figure 3.**
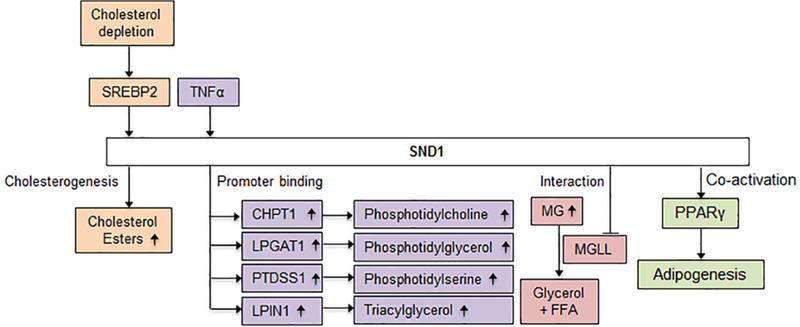
Possible role of SND1 in lipid metabolism. Exposure of human HCC cells to cholesterol-lowering drug or a lipoprotein-deficient medium triggers SREBP2 activation and increases SND1 promoter activity. Studies in rat hepatoma cells show that SND1 overexpression accumulates de novo synthesized cholesteryl esters. SND1 is induced by TNFα and subsequent profiling in human hepatoma cells revealed that SND1 binds to the promoter regions of a group of glycerolipid metabolic genes including CHPT1, LPGAT1, PTDSS1 and LPIN1 involved in the biosynthesis of phophatidylcholine, phosphatidylglycerol, phosphatidylserine and triacylglycerol, respectively. As yet functional consequence of SND1 binding to the promoter of these genes has not been studied. In human HCC cells SND1 interacts with MGLL and results in ubiquitination and proteosomal degradation of MGLL. The increase in monoglyceride (MG) levels is predicted from the known role of MGLL. Studies in mouse adipocytes have shown that SND1 is a co-activator of PPARγ in adipogenesis. SND1: staphylococcal nuclease and tudor domain containing 1; HCC: hepatocellular carcinoma

Glycerolipids are lipids composed of mono, di- or tri- substituted glycerol moieties that are important constituents of biological membranes. Rapid synthesis of lipids is required for generation of biological membranes and facilitating cancer cell proliferation. SND1 induction with TNFα and subsequent profiling of SND1 promoter activity revealed that SND1 regulates a group of glycerolipid metabolic genes including CHPT1, LPGAT1, PTDSS1 and LPIN1 that are involved in biosynthesis of phophatidylcholine, phosphatidylglycerol, phosphatidylserine and triacylglycerol respectively^[58]^. SND1 interacts with and inhibits monoglycerolipid lipase (MGLL)^[41]^, a tumor suppressor that converts monoglycerolipids to glycerols and free fatty acids. Thus, SND1 causes an increase in glycerolipid levels in cells by causing an increase in their synthesis or preventing their catabolism in hepatocytes [Figure 3].

## CONCLUSION

HCC is unique in having defined etiologies, all of which cause chronic inflammation. In addition, altered lipid metabolism in obesity-associated NASH is becoming a major driving force for HCC. It is intriguing that SND1 plays a role in regulating both inflammation and lipid metabolism, and also the hallmarks of cancer by a variety of mechanisms, suggesting that targeting SND1 might be a viable option for HCC. This notion is strengthened by the observation that Alb/SND1 mice develop spontaneous HCC, thus establishing SND1 as a tumor driver^[43]^. SND1 is the only eukaryotic protein with a tudor and SN domains and the quaternary fold can be employed to obtain specific small molecule inhibitors, such as pdTp. The efficacy of pdTp in inhibiting growth of HCC xenografts *in vivo* is exciting and promising. However, this inhibitor is required in high doses to inhibit SND1 and inhibits only the nuclease function and not the nucleic acid binding function. Thus, it is important to identify better analogs of pdTP and develop strategies that can achieve complete inhibition of SND1. Recent success of hepatocyte-specific nanoparticle-delivered siRNA targeting oncogenes in HCC opens up potential of such strategy to inhibit SND1. Genetic deletion studies *in vivo* would provide a clue to the effects such inhibitors could produce. Further in-depth studies using *in vitro* and *in vivo* models are required to better understand the functional attributes of this pleiotropic molecule so that it is efficiently targeted.
